# Drug delivery to retinal photoreceptors

**DOI:** 10.1016/j.drudis.2019.03.004

**Published:** 2019-08

**Authors:** Erico Himawan, Per Ekström, Matej Buzgo, Pieter Gaillard, Einar Stefánsson, Valeria Marigo, Thorsteinn Loftsson, François Paquet-Durand

**Affiliations:** 1 InoCure s.r.o., Prague, Czech Republic; 2 Lund University, Faculty of Medicine, Department of Clinical Sciences Lund, Ophthalmology, Lund, Sweden; 3 2-BBB Medicines BV, Leiden, The Netherlands; 4 Faculty of Medicine, University of Iceland, Reykjavík, Iceland; 5 Department of Life Sciences, University of Modena and Reggio Emilia, Italy; 6 Faculty of Pharmaceutical Sciences, University of Iceland, Reykjavík, Iceland; 7 Institute for Ophthalmic Research, University of Tübingen, Germany

## Abstract

The photoreceptors of the retina are afflicted by diseases that still often lack satisfactory treatment options. Although suitable drugs might be available in some cases, the delivery of these compounds into the eye and across the blood–retinal barrier remains a significant challenge for therapy development. Here, we review the routes of drug administration to the retina and highlight different options for drug delivery to the photoreceptor cells.

## Highlights

• Routes of administration to retinal photoreceptors.
• The blood–retinal barrier as a challenge for photoreceptor drug delivery.
• Review of nanoparticle drug delivery systems used for intraocular applications.
• Perspectives for topical drug delivery to the retina.

## The retina and photoreceptor degeneration

The retina is the light-sensitive, neuronal tissue that sits at the back of the eye. Within the retina, the photoreceptors (i.e., rods and cones) are the primary neurosensory cells that convert light into an electrochemical message that can be interpreted by the central nervous system (CNS). In the human retina, there are ∼120 million rod and ∼6 million cone photoreceptors. Whereas rods are more sensitive to low light (as in night vision), cones are more suited for colour discrimination and visual acuity in daylight, which is generally regarded as the ability to see [1].

Vision loss owing to photoreceptor degeneration is a devastating handicap with far-reaching effects on the quality-of-life of the affected individuals and on society as a whole [2]. Photoreceptor degeneration occurs in etiologically different diseases, with important causes being diabetic retinopathy [3], age-related macular degeneration (AMD) [4] and hereditary retinal degeneration [5]. Because photoreceptors are post-mitotic neurons they will not be replaced upon degeneration, which thus permanently reduces the ability of the retina to capture light, leading to progressive severe visual impairment and, frequently, blindness. In hereditary diseases of the retina, the causative mutations often affect rods. However, when diseased rods degenerate and die this will lead to a secondary loss of cones, even if these are genetically unaffected [5]. A similar mechanism has been proposed for AMD where initial rod loss in central parts of the retina can entrain cone loss in the macula [6].

## The problem of retinal drug delivery

**Figure 1:**
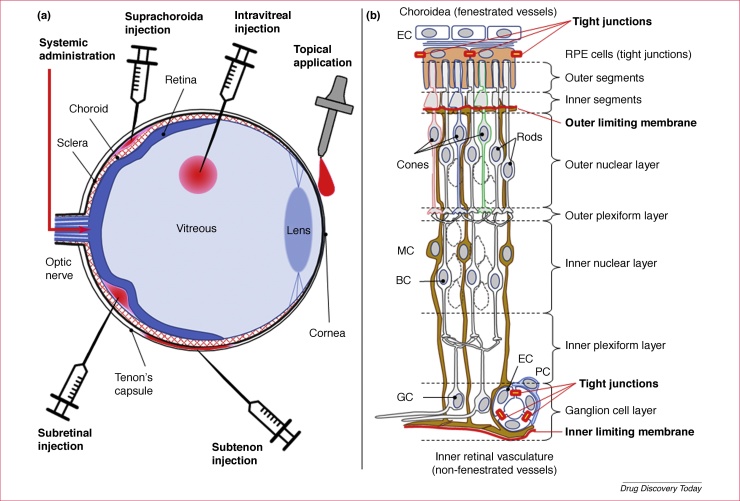
Routes of administration for drugs targeted to the photoreceptors of the retina − illustration of the blood–retinal barrier. **(a)** Diagrammatic cross-section through an eye, illustrating different routes for administration of drugs to the retina. The ocular cross-section shows on the posterior side (left) the optic nerve, the Tenon capsule surrounding the eye, the sclera, the choroidal vasculature (choroid) and the retina (shown in blue). The anterior side (right) shows the vitreous body, the lens and the cornea. The application routes highlighted are topical, intravitreal, subtenon, suprachoroidal and subretinal injections, as well as systemic administration via the general blood circulation. **(b)** Idealised cross-section through the retina displaying the choroid and retinal pigment epithelium (RPE; top), the outer and inner nuclear layers, as well as the ganglion cell (GC) layer (bottom). The components of the outer and inner blood–retinal barrier, including the outer and inner limiting membranes, are highlighted in red. Note that the retinal structure has been simplified for clarity and that not all retinal cell types are shown. Abbreviations: BC, bipolar cells; EC, endothelial cell; MC, Müller glial cell; PC, pericyte.

The research on how to counteract the degenerative retinal diseases is extensive [7]. However, in addition to developing compounds that interact with relevant molecular targets, the field must also consider how to deliver new drugs to the retina. The retina is protected by the blood–retinal barrier (BRB) [8], which by engulfing all retinal blood vessels maintains retinal homeostasis and shields from, for example, blood-borne toxins or infectious agents. The BRB consists of two different barriers: (i) the outer BRB, which is formed by endothelial cells lining the choroidal vasculature (fenestrated blood vessels) and tight-junction-coupled retinal pigment epithelial (RPE) cells; and (ii) the inner BRB, which is formed by endothelial cells (non-fenestrated vessels) in conjunction with pericytes, astrocytes and Müller glial cells. The inner BRB is analogous to the blood–brain barrier (BBB), perhaps with the exception of Müller glia. The Müller glial cells could be of particular importance for the BRB because they form the outer and inner limiting membranes, which provide additional shielding of the neuroretina towards the RPE cells and the vitreous humor, respectively (Fig. 1). Although the BRB is essential for the protection and viability of the retina, it can also prevent access of therapeutic compounds and, to date, relatively few studies have addressed this problem in a comprehensive way.

In principle, there are several routes available for administration of drugs to the photoreceptors of the retina (Fig. 1). A systemic administration of drugs intended for the retinal photoreceptors is potentially possible when using a drug delivery system (DDS) targeting the CNS, as has been recently demonstrated with liposomes conjugated with polyethylene glycol (PEG) chains, which in turn carry a glutathione (GSH) molecule [9]. This route of administration could, however, in practice be limited by systemic side-effects of the encapsulated compound (depending on the drug properties and the location of the drug target) and cost of goods. An elegant combination of delivery technologies to address these potential issues could be the use of a suprachoroidal injection technique, which effectively delivers the drug (or drug nanoparticle) with precise access to the choroid and the retina where the diseases manifest. Several clinical studies employing this technique are currently underway [10].

Another approach for local delivery directly to the photoreceptors is subretinal injection between the retinal pigment epithelium (RPE) and the outer segments of the photoreceptors. Although, to date, this is the route of choice for retinal gene therapy [11], the procedure will inherently result in retinal detachment, which is detrimental to retinal viability *per se*. Where frequent re-administrations are required, the damage resulting from subretinal injections could thus quickly offset any beneficial effects of the delivered drug.

Other routes of administration (Fig. 1) that will deliver a drug physically close to the retina include subtenon injection (i.e., injection into the capsule of Tenon) [12] and intravitreal injection [13]. Because the inner retinal blood vessels are reached from the vitreous side, the latter is the route of choice for antiangiogenic drugs used for the treatment of AMD and diabetic macular oedema, even though the procedure carries a risk of serious intraocular inflammation [14]. Hence, less invasive routes of delivery, such as topical administration, might be preferred. Regardless of the chosen route, the drug formulation and delivery system used will be crucial for successful treatment development.

## Nanoparticles for intraocular drug delivery

Nanoparticles, irrespective of their type and material, could constitute an ideal DDS for several reasons: protecting the cargo, sustained release, improved drug localisation and enhanced drug efficacy [15]. Although their use as a DDS is more common in ocular applications, nanoparticles can also act as the active component of the therapy, including for retinal disease, where nanoparticles such as nanoceria have shown promise [16]. This review, however, will mainly be focused on their uses as ocular DDS.

One of the main reasons to use a DDS is because some suitable drug or molecule can be negatively affected by the environmental conditions. This is especially true for sensitive cargo such as DNA and RNA for therapeutic gene therapy. However, when DNA plasmids are incorporated into polyplexes of cationic polymers, they can be formed into nanoparticles, which can protect them from the nuclease-rich environment and also enhance cellular uptake [17]. Moreover, improved cellular uptake of the drug molecule can be coupled with sustained delivery. For instance, Suen and Chau have shown that, by using folate to decorate PEG-b-PCL nanoparticles for triamcinolone acetonide encapsulation, enhanced uptake by RPE cells was observed and the controlled release of the cargo drug resulted in prolonged antiangiogenic gene expression [18].

A variety of different DDS have been designed to enhance drug delivery to the posterior segment of the eye, including to the retinal photoreceptors (Table 1), yet the different DDS need to be tested based on the physicochemical characteristics of the drug to be delivered. Material properties will determine much of the carrier characteristics of the nanoparticle. The more advanced generation of nanoparticles for DDS might include material that can change its properties when given a specific stimulus. For instance, light-responsive polymers can be used to design nanoparticle depots to deliver cargo after a brief, low-power light exposure – a noninvasive way of triggering intraocular drug release [19]. Remarkably, these nanoparticles can deliver cargo to the retina in response to UV exposure up to 30 weeks post-injection. Such controlled-release designs could be beneficial for prolonging intravitreal injection intervals.

**Table 1:** Examples of nanoparticle application for intraocular drug delivery

Adm. route	DDS	Carrier composition	Cargo	Effect
Topical	Polymeric nanoparticles [40]	PLGA and PEG	Dorzolamide	• 2x increase in therapeutic duration • 35% more effect in intraocular pressure (IOP) decrease
	Solid lipid nanoparticles (SLN) [41]	Stearic acid, Epikuron™ 200, sodium taurocholate	Trobramycin	• Higher drug conc. in all ocular tissues • Greater bactericidal activity against intracellular *Pseudomonas aeruginosa*
	Solubilising nanoparticles [23]	γ-Cyclodextrin	Dexamethasone, dorzolamide	• Enhanced residence time on the eye surface • Enhanced solubility in the tear fluid • Up to about tenfold enhancement in bioavailability
	Niosomes [42]	Span 60, cholesterol, ethanol	Prednisolone sodium phosphate	• Ocular bioavailability 1.75x greater • Healing time 2x faster • Significantly less IOP elevation as side effect
	Liposomes [43]	Soybean phosphatidylcholine, cholesterol	Timolol maleate	• Longer retention time on corneal surface • Faster reduction in IOP

Intravitreal	Polymeric nanoparticles [19]	Light-sensitive polymers	Nintedanib (BIBF1120)	• Carrier can release cargo up to 30 weeks post-intravitreal injection • Release can be controlled by irradiation
	SLN [44]	Precirol^®^ ATO5, DOTAP, Tween^®^ 80, protamine, dextran	Human Rs1 gene	• Higher transfection level in photoreceptors • Significant structural improvement in retinal layer
	Niosomes [45]	Cationic lipid, squalene, polysorbate 80	pCMSEGFP plasmid	• Successfully transfected HEK-293 and ARPE-19 cells without affecting viability
	Liposomes [46]	Cholesterol derivatives, PEG, l-α-phosphatidylcholine	Doxorubicin	• Significantly improve drug stability • More drug cargo delivered to retina

Systemic	Polymeric nanoparticles [47]	PLGA, tripeptide adhesion motif Arg-Gly-Asp (RGD)	Flt23k plasmids	• RGD modified nanoparticles localised in choroidal neovascularization (CNV) lesions • No secondary CNV lesions in treated mice • Vision function is restored exclusively after treatment
	SLN [41]	Stearic acid, Epikuron™ 200, sodium taurocholate	Trobramycin	• Using SLN, drug concentration after 1–3 h from intravenous administration is 10 x higher than those without SLN
	Liposomes [48]	Soybean phosphatidylcholine, cholesterol, glutathione-PEG	Methylprednisolone hemisuccinate	• Improved efficacy in a model of ocular inflammation • Protection from experimental autoimmune uveitis
	Liposomes [9]	Soybean phosphatidylcholine, cholesterol, glutathione-PEG	Cyclic nucleotide analogue CN03	• Neuroprotection of photoreceptors in three different animal models for retinal degeneration • Preservation of cone photoreceptor function

Nanoparticle surface modification can also play a significant part in developing a better DDS. It is understood that vitreal charge, which results mostly from the presence of hyaluronic acid in the vitreous humor, can be significant in hindering nanoparticle movement 20, 21, 22. Accordingly, cationic nanoparticles will have more difficulty in diffusing through the vitreous humor. However, liposomal surface modifications, such as PEGylation, could help to improve their intravitreal mobility [21].

## Approaches for topical delivery to the retina

Topical drug delivery to the retina is hampered by three major obstacles [23].

• The aqueous tear fluid and the exterior mucus layer. Only dissolved drug molecules can permeate from the surface into the eye and, thus, for effective delivery, topically applied drug must possess sufficient aqueous solubility. Furthermore, although drug transporters have been located in the eye, drug molecules are mainly transported from the eye surface into the eye by passive diffusion where the concentration gradient of dissolved drug molecules is the driving force.
• The short contact time of dissolved drug molecules with the eye surface. In humans the volume of the aqueous tear fluid is ∼7 μl and the normal tear turnover is about 1.2 μl/min [24]. Thus, the precorneal half-life of the dissolved drug is only ∼3 min.
• The lipophilic membrane barriers (i.e., cornea and conjunctiva). These dictate that the dissolved drug molecules must possess sufficient lipophilicity to be able to permeate the lipophilic membrane barriers.

From the above it is obvious that successful delivery from the tear film into the eye requires a drug to have adequate aqueous solubility and lipophilicity, as well as to be able to maintain high concentrations in the tear film for a sufficient amount of time. Yet, most ophthalmic vehicles and DDS for topical drug delivery still only address one of these three permeation obstacles. For example, vehicles like hydrogels and devices like contact lenses only increase the drug residence time on the eye surface but, in general, do not increase the drug concentration gradient over the membrane barriers. Likewise, conventional nanoparticles generally only increase the drug residence time. Formation of hydrophilic prodrugs of lipophilic drugs, or of lipophilic prodrugs of hydrophilic drugs, addresses only one obstacle (i.e., solubility in the tear fluid or permeation through lipophilic membrane, respectively) while intensifying the other. Then there are chemical penetration enhancers like quaternary ammonium compounds and cell-penetrating peptides that enhance drug delivery by temporarily damaging the membrane barriers to make them more permeable 25, 26. However, ideally, the vehicle or DDS should enhance drug permeation from the surface into the eye without affecting the ocular permeation barriers. Drug delivery to the posterior segment of the eye has been the subject of a recent review [27].

Two main permeation routes of topically applied drugs into the posterior segment of the eye have been proposed: the corneal route (i.e., cornea → aqueous humor → intraocular tissues → posterior segment) and the scleral route (i.e., conjunctiva → sclera → choroid/retinal pigment epithelium → vitreous humor) [28]. Studies in rabbits indicate that, for drugs like dexamethasone, the scleral route might be dominating [29].

**Figure 2:**
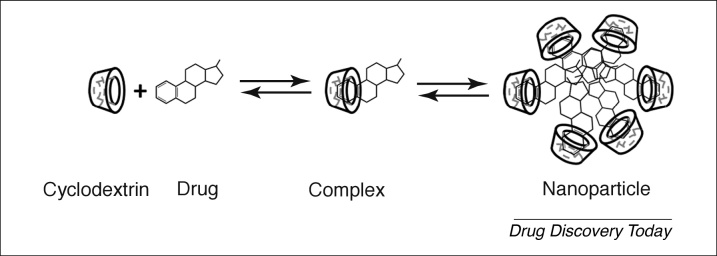
Formation of the solubilising drug–cyclodextrin nanoparticle. The cyclodextrin molecules, which are frequently referred to as host molecules, are displayed as a cup-like structure, which initially forms a complex with individual host molecules. The host shown here represents an idealised dexamethasone molecule. At higher concentrations, drug–cyclodextrin complexes aggregate to form larger nanoparticles that can be administered topically to the eye.

In an effort to simultaneously address all three obstacles of topical drug delivery into the eye, solubilising nanoparticles were developed that not only increase the drug residence time on the eye surface but also its solubility in the aqueous tear fluid. These solubilising nanoparticles consist of drug–cyclodextrin complexes that have somewhat limited solubility in the aqueous tear fluid but are readily dissolved upon media dilution (Fig. 2). At low cyclodextrin concentrations the aqueous media is a clear solution but when the cyclodextrin concentration is increased the solution becomes milky owing to the formation of nano- and/or micro-suspension. Cyclodextrins are extremely hydrophilic oligosaccharides that are metabolised in the gastrointestinal tract. They solubilise lipophilic drugs that are poorly soluble in aqueous media, such as the tear fluid, without affecting the ability of the drug molecules to permeate lipophilic membrane barriers, like the conjunctiva and cornea. Certain cyclodextrins form complexes that have only limited solubility in water and, thus, can form nano- and micro-suspensions. After administration in aqueous eyedrops the particles adhere to the eye surface and dissociate upon dilution of the tear fluid keeping it saturated with the dissolved drug for >7 h after single application [23]. The result is a greater than tenfold increase in topical bioavailability of small lipophilic drugs.

Another observation is that, although solubilising nanoparticles enhance drug delivery from the eye surface into the eye via the corneal and scleral routes, the scleral route appears to be the dominating route to the retina. Table 2 shows, as an example, the dexamethasone concentration in various eye tissues 2 h after administration of aqueous eyedrops containing dexamethasone–cyclodextrin microsuspension. According to Fick’s first law, passive diffusion of drug molecules from the eye surface into the posterior segment is driven by the drug concentration gradient. The concentration levels, in particular the low dexamethasone levels in the vitreous humor, indicate that the drug is mainly delivered to the retina via the scleral route, although some is certainly reaching the retina via the corneal route.

**Table 2:** Concentration (mean ± SEM, n = 6) of dexamethasone in various eye tissues 2 h after topical administration of 50 μl of aqueous 1.5% (w/v) dexamethasone eyedrops containing dexamethasone–γ-cyclodextrin complex microsuspension to rabbits

The drug was administered to the left eye and the drug concentration determined in both eyes. The term topical denotes how much the topical absorption contributed to the dexamethasone level in a given tissue (i.e., the difference of the left and right eye concentrations). The plasma concentration at 2 h was determined to be 35 ± 16 ng/ml [27].

## Liposomes for systemic and intraocular delivery

Another option for helping compounds to cross the BRB could be to encapsulate them into suitable vehicles, with properties that favour crossing the barrier and thus bringing the cargo release closer to the target. This could be accomplished by PEGylated liposomes, in which the PEG molecules carry a GSH molecule at their external endings. In preclinical studies, this nanoscale liposomal carrier was previously shown to improve delivery of the drug doxorubicin to the brain [30]. How exactly the liposomal cargo is ultimately transported across the BBB is not entirely clear but the liposomal uptake appears to be an active process that is based on a GSH- and cytoskeletal-rearrangement-dependent endocytosis of the liposome [31]. Because the BBB and the BRB have essentially the same structural features 32, 33, this liposomal DDS could also facilitate drug delivery to the retina and its photoreceptors. Accordingly, it was demonstrated that systemic use of the GSH-conjugated, PEGylated liposomes could deliver fluorescein tracer as well as the cyclic nucleotide analogue CN03 to photoreceptor cells in the mouse [9]. Although the compound CN03 alone, without liposomal formulation, was unable to produce significant photoreceptor protection, with liposomal encapsulation CN03 administration resulted in significant photoreceptor protection and preservation of retinal function in different animal models for hereditary retinal degeneration [9].

Other than systemic administration, liposomes can also be used for direct drug delivery to the eye using intravitreal injection [34]. This approach was, for instance, used for the encapsulation of recoverin – a protein important for the phototransduction cascade in photoreceptors. When non-PEGylated liposomes (i.e., lipid vesicles) were injected into the vitreous body of the eye, they demonstrated preferential cargo delivery to the photoreceptors, resulting in an increased sensitivity of rod photoreceptors to light [35]. This study provided a proof-of-concept for the liposomal delivery of whole proteins to retinal photoreceptors, a finding that could potentially also be used for the transfer of therapeutic proteins. Taken together, liposomes have demonstrated their potential for photoreceptor delivery of low molecular weight compounds and high molecular weight proteins with strong promise for future drug delivery development.

## Modulation of the BRB

An entirely different possibility to mediate drug transfer across the BRB might be to utilise a temporary opening by targeted downregulation of crucial BRB constituents. This has been successfully achieved by the temporary siRNA-mediated knockdown of a key component of the tight junctions that constitute the BRB [36]. In this scenario, a drug applied via the bloodstream, within a defined time slot after siRNA-mediated knockdown of the BRB component, would be taken up locally in the retina.

Several recent studies employed this approach targeting claudin-5, a component of tight junctions and mediating cell–cell coupling in the BRB. Claudin-5 knockdown via siRNA injected hydrodynamically into the tail vein of rats led to a temporary breakdown of the BRB lasting for ∼72 h post-inoculation [37], enabling an efficient transfer of therapeutic agents up to a molecular weight of 500–1000 Da. When suppression of claudin-5 expression was combined with the co-suppression of another tight-junction protein called occludin, the barrier permeability was increased and allowed transfer of compounds up to a molecular weight of ∼4 kDa [38]. In animal studies this approach appeared to be well tolerated. Although these results with the BRB-modulating approach appear highly promising for single applications, it remains doubtful whether this approach would be useful in the context of a chronic disease, which would require frequent reapplications. This issue could potentially be resolved with a gene-therapy approach, in which an inducible siRNA construct delivered to the retina via a viral vector would be permanently transduced but activated only with doxycycline administration [39]. It is of note that transient downregulation of the BRB could carry a risk for infection or inflammatory response, which might need to be investigated in further studies.

## Concluding remarks

The past decade has seen a dramatic development, with many new materials, designs and technologies becoming available, that opens unprecedented possibilities for ocular drug delivery. Nevertheless, drug delivery to the neuroretina, and even more so to the retinal photoreceptors, still has inherent and important challenges, including an estimation of which DDS is most suitable for the drug and for the target as such, in accordance with the requirements from the medicinal drug and product authorities. This should not reflect on the scaling up of successful experimental findings to meet good manufacturing practice (GMP) conditions, which demand a tighter intersectoral collaboration between academia and industry, to bring together the required knowledge and expertise. Hence, future research into design and development of drugs for retinal diseases will have to address the delivery aspects in a comprehensive way, and from a very early point on during compound development.
